# Neurodevelopmental regression Due to PLA2G6-associated neurodegeneration despite normal brain MRI: a case report

**DOI:** 10.3389/fped.2026.1869053

**Published:** 2026-06-30

**Authors:** Andrea Milena Rodríguez-Guerrero, Alexandra Romero Valdez, Marjorie Rodríguez-Guerrero, Jhoana Piedra Duran, Eduardo Flores, Jorge Vasconez-Gonzalez, Juan S. Izquierdo-Condoy, Esteban Ortiz-Prado

**Affiliations:** 1 Universidad de Las Américas, Quito, Ecuador; 2 Departamento de Pediatría, Hospital Vicente Corral Moscoso, Cuenca, Ecuador; 3 Interno Rotativo de Medicina, Hospital Vicente Corral Moscoso, Cuenca, Ecuador; 4 One Health Research Group, Universidad de Las Américas, Quito, Ecuador

**Keywords:** brain iron accumulation disorders, infantile neuroaxonal dystrophy, neurodegenerative disorder, neurodevelopmental regression, PLA2G6

## Abstract

**Background:**

Infantile neuroaxonal dystrophy is a rare autosomal recessive neurodegenerative disorder within the spectrum of PLA2G6-associated neurodegeneration. It is typically characterized by early psychomotor regression, progressive motor impairment, bulbar dysfunction, and characteristic neuroimaging abnormalities, including cerebellar atrophy and, in some cases, brain iron accumulation.

**Case report:**

We report the case of a 4-year–11-month-old girl with progressive neurodevelopmental regression beginning at approximately 18 months of age. The patient presented with loss of previously acquired motor abilities, absence of verbal language, marked hypotonia, preserved deep tendon reflexes, and progressive feeding difficulties. Laboratory evaluation was largely unremarkable, except for persistent isolated elevation of aspartate aminotransferase (AST). Brain MRI showed no overt structural or signal abnormalities despite advanced neurological impairment. Electroencephalography (EEG) did not reveal epileptiform activity. Given the progressive course and suspicion of an underlying neurodegenerative disorder, whole-exome sequencing was performed and identified a homozygous PLA2G6 variant, supporting the diagnosis of PLA2G6-associated neurodegeneration. During follow-up, the patient developed dysphagia and grade II gastroesophageal reflux, requiring gastrostomy placement and Nissen fundoplication.

**Conclusion:**

This case highlights the importance of early molecular diagnosis in pediatric regression syndromes and emphasizes that normal neuroimaging does not exclude PLA2G6-associated neurodegeneration, particularly in atypical presentations. Early recognition enables timely multidisciplinary management, prognostic assessment, and appropriate genetic counseling for the family.

## 1 Introduction

Infantile neuroaxonal dystrophy (INAD) is a rare autosomal recessive neurodegenerative disorder first described by Seitelberger in 1952 (1, 2). It is currently recognized as part of the spectrum of PLA2G6-associated neurodegeneration and belongs to the broader group of disorders known as neurodegeneration with brain iron accumulation (NBIA) (2). Although its true prevalence remains uncertain, INAD is estimated to affect approximately 1 in 1,000,000 children worldwide (3).

INAD is caused by pathogenic variants in the PLA2G6 gene, located on chromosome 22q13, which encodes a calcium-independent phospholipase A2. This enzyme is involved in the hydrolysis of glycerophospholipids, a process essential for the remodeling of axonal and synaptic phospholipid membranes. Disruption of this pathway impairs membrane homeostasis and reduces the ability of neurons to repair oxidative damage, ultimately contributing to progressive neuroaxonal degeneration (1). Clinically, INAD typically presents between 6 months and 3 years of age with developmental arrest or psychomotor regression, truncal hypotonia, progressive motor impairment, and eventual spastic tetraparesis. Additional manifestations may include gait disturbance, loss of vocalization, optic atrophy, bulbar dysfunction, and seizures (4).

Brain magnetic resonance imaging (MRI) plays an important role in the diagnostic evaluation of INAD and may reveal iron accumulation in the basal ganglia, although this finding can be absent in the early stages of the disease. Other commonly reported neuroimaging features include cortical and cerebellar atrophy, thinning of the optic chiasm, and white matter hyperintensities (5). However, the absence of typical MRI abnormalities does not exclude PLA2G6-associated neurodegeneration, particularly in early or atypical presentations. INAD is associated with a poor prognosis, with an estimated mean age at death of approximately 9.9 years, most commonly due to respiratory failure secondary to bulbar involvement (1).

In this report, we describe a child with progressive neurodevelopmental regression, bulbar dysfunction, and a homozygous *PLA2G6* variant, whose conventional brain MRI showed no overt abnormalities despite advanced clinical manifestations. This case highlights the diagnostic value of molecular testing in pediatric regression syndromes and emphasizes that normal neuroimaging should not preclude consideration of PLA2G6-associated neurodegeneration.

Previous reports have described initially normal or nonspecific neuroimaging findings in patients with PLA2G6-associated neurodegeneration. For instance, Wang et al. reported a child with early neurodevelopmental regression whose initial brain MRI was unremarkable; however, follow-up imaging later demonstrated cerebellar atrophy, suggesting progression toward the classical radiological phenotype of infantile neuroaxonal dystrophy (INAD) (6).

In contrast, our patient presented with advanced clinical manifestations, including severe motor regression, absence of verbal language, marked hypotonia, dysphagia, and significant functional impairment, despite persistently normal findings on conventional brain MRI. This clinicoradiological dissociation is particularly relevant, as it demonstrates that severe neurological deterioration may precede detectable structural abnormalities on routine neuroimaging.

Therefore, pediatricians should recognize that a normal MRI does not exclude PLA2G6-associated neurodegeneration in children with progressive developmental regression. Early genomic testing should be strongly considered even in the absence of characteristic radiological findings, as delayed diagnosis may postpone multidisciplinary management, prognostic counseling, and genetic assessment of the family.

## 2 Case report

We report the case of a 4-year–11-month-old female patient with no significant personal or family medical history, who was referred for evaluation due to progressive neurodevelopmental regression, primarily characterized by the loss of previously acquired motor abilities.

The patient was born following an uncomplicated pregnancy, with adequate prenatal care initiated during the third month of gestation. She was delivered by cesarean section at 38 weeks of gestation due to severe oligohydramnios. Her neonatal condition was appropriate, with Apgar scores of 8 and 9 at 1 and 5 min, respectively. Birth weight was 3,100 g, length was 49 cm, and head circumference was 34.5 cm. No neonatal complications were reported. Newborn metabolic and hearing screening results were normal. She was the only child of healthy, non-consanguineous parents; the mother was 20 years old and the father was 24 years old at the time of evaluation, both without relevant medical history.

Regarding psychomotor development, the patient achieved head control at 4 months and independent sitting at 6 months. However, subtle motor delay was retrospectively noted, as she did not crawl and never achieved independent walking, only ambulation with support. In the language domain, early development was limited, with initial production of simple words such as “mama” and “papa,” followed by subsequent language regression. At the time of evaluation, she communicated only through crying and moaning. She had not achieved age-appropriate bladder or bowel control.

Although early neurodevelopmental milestones were partially attained during the first months of life, clear developmental regression became evident at approximately 18 months of age. This regression was characterized by progressive loss of supported ambulation, deterioration of global motor function, and loss of expressive language. Neurological examination revealed marked generalized hypotonia (Figure 1), reduced spontaneous motor activity, and decreased overall muscle strength, with preserved deep tendon reflexes. Reflexes remained preserved throughout the follow-up period. No sensory abnormalities were identified. The remainder of the physical examination was unremarkable.

**Figure 1:**
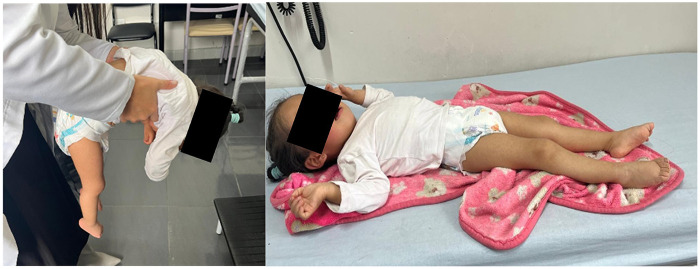
Clinical evidence of marked generalized hypotonia.

Initial laboratory studies showed a blood glucose level of 98 mg/dL, urea 21 mg/dL, uric acid 4 mg/dL, and creatinine 0.43 mg/dL. Liver function tests revealed a persistent elevation of aspartate aminotransferase (AST), with an initial value of 109.57 U/L and a subsequent value of 139 U/L. In contrast, alanine aminotransferase (ALT) remained within normal ranges on follow-up testing, with values of 23.7 U/L and 16.7 U/L. Gamma-glutamyl transferase (GGT) levels were within normal limits. Complete blood count showed a leukocyte count of 5.07 × 10^3^/µL and a hemoglobin level of 14.6 g/dL. Thyroid function was normal, with a thyroid-stimulating hormone (TSH) level of 1.18 µIU/mL.

Given the progressive neurodevelopmental regression and the suspicion of an underlying genetic neurodegenerative disorder, whole-exome sequencing was performed on June 12, 2023. The analysis identified a homozygous variant in the PLA2G6 gene, resulting in the amino acid substitution p.Pro660Ser (Table 1). This finding supported the diagnosis of PLA2G6-associated neurodegeneration within the clinical spectrum of infantile neuroaxonal dystrophy. Additional variants were identified in TTN and SYNE1; however, based on available in silico predictions and clinical correlation, these variants were not considered to account for the patient's phenotype.

**Table 1:** In silico pathogenicity prediction of identified genetic variants.

Gene	Amino acid change	MutationTaster	SIFT	PolyPhen-2	CADD
*PLA2G6*	p.Pro660Ser	D	D	D	27.7
*TTN*	p.Leu12219Val	P	NA	NA	0.001
*SYNE1*	p.Phe2938Leu	N	T	B	13.79

Brain magnetic resonance imaging performed on 02/06/2023 demonstrated preserved global cerebral and cerebellar morphology without evidence of cerebellar atrophy, cortical volume loss, ventricular enlargement, or focal white matter abnormalities (Figure 2). Basal ganglia structures appeared symmetric without evident signal alterations on conventional sequences. No mass effect, hemorrhage, hydrocephalus, or midline shift was identified. Sagittal imaging showed preserved morphology of the corpus callosum, brainstem, and cerebellar vermis. Overall, no overt structural or signal abnormalities suggestive of advanced neurodegeneration were detected on routine MRI sequences.

**Figure 2:**
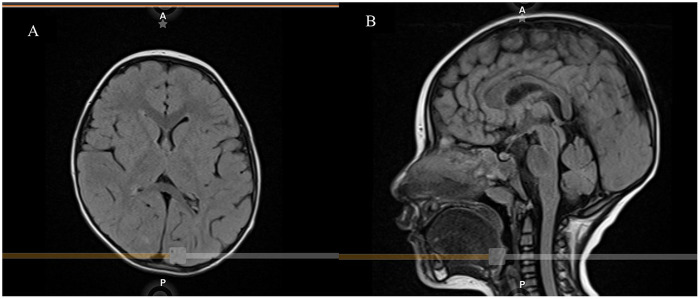
Brain MRI without overt structural or signal abnormalities. Axial section **(A)**, transverse section **(B)** Study shows no abnormalities. Cerebral parenchyma without morphological alterations; white and gray matter are consistent with what is expected for the patient's age. There is no ventricular dilation or midline shift. (The MRI was performed at 3 years and 6 months of age).

However, susceptibility-sensitive sequences such as susceptibility-weighted imaging (SWI) or T2*-weighted gradient-echo imaging were not available; therefore, subtle iron deposition within the basal ganglia could not be definitively excluded.

Representative axial brain MRI images demonstrated preserved cerebral parenchymal morphology, normal ventricular size, and no evidence of cerebellar atrophy, white matter signal abnormalities, basal ganglia involvement, ventricular dilation, midline shift, hemorrhage, or venous thrombosis. Magnetic resonance imaging of the cervical, thoracic, and lumbar spine, performed on June 21, 2023, did not reveal significant abnormalities. The facet joints showed no evidence of arthrosis or disjunction, the neural foramina were preserved, and the paravertebral musculature appeared normal.

An electroencephalogram performed in 2024 showed no epileptiform activity. During follow-up, the patient exhibited progressive neurological deterioration, with marked neurodevelopmental regression, absence of verbal language, and severe limitation of spontaneous motor activity. She remained alert, although her baseline neurological impairment persisted.Initially, the patient was treated with baclofen due to suspected limb spasticity. However, after reassessment by pediatric neurology and considering the predominance of hypotonia as well as the overall risk–benefit profile, baclofen was discontinued.

As the disease progressed, the patient developed feeding difficulties. An upper gastrointestinal series demonstrated impaired swallowing mechanics associated with grade II gastroesophageal reflux. Given the risk of aspiration and nutritional compromise, surgical intervention was indicated. The patient underwent gastrostomy placement and Nissen fundoplication, both of which were completed without immediate complications. Following surgery, she remained under multidisciplinary follow-up, receiving enteral feeding via gastrostomy, with ongoing monitoring of her neurological status, nutritional condition, swallowing safety, and respiratory risk.

## 3 Discussion

The present case describes a child with progressive neurodevelopmental regression, loss of previously acquired motor and language abilities, feeding difficulties, and marked hypotonia with preserved deep tendon reflexes. Although early milestones were partially achieved during the first months of life, subtle motor delay was retrospectively evident, as the patient did not crawl and never achieved independent walking. The subsequent onset of clear regression at approximately 18 months of age, together with progressive global motor deterioration, raised the suspicion of an underlying genetically determined neurodegenerative disorder, particularly within the spectrum of PLA2G6-associated neurodegeneration (Figure 3).

**Figure 3:**
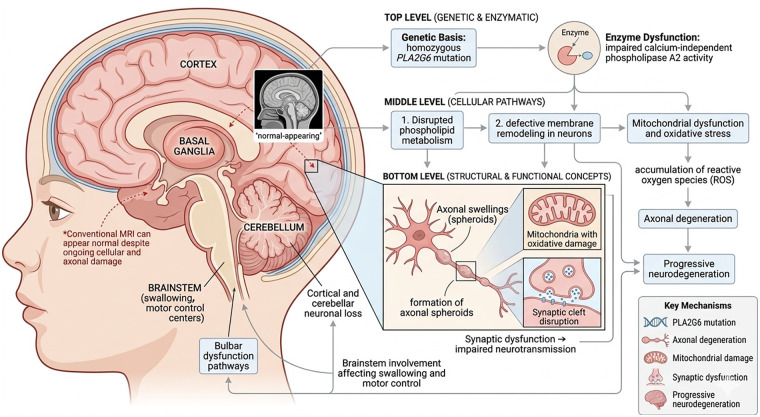
Pathophysiology of infantile neuroaxonal dystrophy associated with *PLA2G6.*

The clinical presentation was consistent with previously described features of infantile neuroaxonal dystrophy (INAD). The diagnosis should be considered in children presenting with psychomotor arrest or regression associated with marked hypotonia during infancy, or with progressive difficulty walking during the second year of life (7). Aicardi and Castelein also reported cases in which symptoms began at 12 months of age or later, supporting the clinical variability of disease onset (8). In the present patient, the combination of early motor delay, progressive regression, loss of expressive language, and bulbar involvement was strongly suggestive of a neurodegenerative disorder. The identification of a homozygous *PLA2G6* variant further supported the diagnosis of PLA2G6-associated neurodegeneration within the clinical spectrum of INAD.

One relevant feature of this case was the persistence of preserved deep tendon reflexes. Classical descriptions of INAD often report progression from hypotonia toward pyramidal signs and hyperreflexia; however, the neurological phenotype may vary across patients and disease stages. Therefore, preserved reflexes should not exclude the diagnosis when the broader clinical picture is compatible with PLA2G6-associated neurodegeneration. In this patient, the predominance of hypotonia, severe motor impairment, and progressive functional decline illustrates the phenotypic heterogeneity of this disorder.

A particularly important aspect of this case is the absence of typical neuroimaging abnormalities. Brain MRI was reported as normal despite advanced clinical manifestations, without evident cerebellar atrophy, basal ganglia involvement, white matter signal abnormalities, or structural changes. This finding contrasts with the classical neuroimaging pattern described in INAD, in which cerebellar atrophy is frequently reported and may be accompanied by optic pathway involvement, white matter abnormalities, or iron deposition in the basal ganglia. Although radiological abnormalities may be subtle or absent in early disease stages, a completely normal conventional MRI at this degree of clinical progression represents a diagnostic challenge and may suggest an atypical or imaging-negative presentation within the PLA2G6-associated neurodegeneration spectrum. In our case, due to the unavailability of susceptibility-sensitive sequences such as susceptibility-weighted imaging (SWI) or T2*-weighted gradient-echo imaging, the presence of subtle iron deposition in the basal ganglia could not be definitively excluded. Nevertheless, cases have been described in which iron deposition is absent. For example, Illingworth et al. reported three cases in which no iron deposition was observed on MRI, although cerebellar atrophy was evident (9). Similarly, Menicucci et al., in a cohort study including 14 patients, reported absence of cerebellar atrophy in two patients, while iron deposition was present in only half of the cohort (*n* = 7) (10).

This discrepancy reinforces the need for longitudinal neuroimaging follow-up and expert neuroradiological review. Follow-up MRI should ideally include susceptibility-sensitive sequences, such as susceptibility-weighted imaging or T2*, to improve detection of subtle iron deposition that may not be evident on conventional sequences. Therefore, this case underscores the limitations of relying exclusively on neuroimaging findings for diagnosis and supports the early use of molecular testing in children with unexplained neurodevelopmental regression.

Historically, the diagnosis of INAD relied on histopathological demonstration of dystrophic axons. However, the identification of pathogenic variants in *PLA2G6* as the molecular basis of INAD has transformed the diagnostic approach and enabled earlier and more accurate recognition of the disorder (11, 12). *PLA2G6* encodes calcium-independent phospholipase A2, an enzyme involved in phospholipid remodeling and membrane homeostasis. Polster et al. suggested that *PLA2G6* plays a role in neuronal proliferation during brain development and in neuronal maturation within the cortical plate and rhombencephalon (13). In murine models, loss of this enzyme has been associated with impaired protein degradation pathways, disrupted axonal membrane homeostasis, and progressive age-dependent neurological deterioration (14).

From a clinical perspective, the identification of a *PLA2G6* variant has important diagnostic, prognostic, and genetic counseling implications. Given the autosomal recessive inheritance pattern of INAD, parental carrier testing and segregation analysis are important to confirm inheritance and refine recurrence risk counseling. Although whole-exome sequencing identified a homozygous PLA2G6 p.Pro660Ser variant consistent with the patient's clinical phenotype, additional molecular characterization was limited. Parental genetic testing and segregation analysis were not performed; therefore, inheritance of the variant could not be molecularly confirmed. Likewise, formal variant classification according to ACMG/AMP criteria was not available at the time of analysis. The pathogenic relevance of the variant was interpreted primarily based on the clinical presentation, homozygous state, rarity, and support in silico pathogenicity predictions. These limitations should be considered when interpreting the genotype–phenotype correlation described in this report. When biallelic pathogenic or likely pathogenic variants are confirmed, the recurrence risk for future pregnancies is 25% (12). Therefore, genetic counseling should be offered to the family as part of comprehensive care.

The patient's phenotype also aligns with common manifestations reported in the literature. Gross motor regression and language impairment are among the most frequent early clinical features (4). Visual abnormalities, including strabismus, pendular nystagmus, disorganized eye movements, optic atrophy, visual loss, and seizures, have also been described (15, 16). Although seizures and evident visual abnormalities were not documented in this patient, their absence does not exclude the diagnosis, given the broad clinical heterogeneity of PLA2G6-associated disorders.

Another clinically relevant feature was the presence of dysphagia with impaired swallowing mechanics and grade II gastroesophageal reflux, which required gastrostomy placement and Nissen fundoplication. Bulbar dysfunction, gastrointestinal complications, nutritional impairment, and aspiration risk are part of the natural course of INAD and often require multidisciplinary supportive management (1, 16, 17). In this context, enteral feeding was indicated to reduce aspiration risk, support nutritional status, and improve overall clinical care. As no disease-modifying treatment is currently available, management remains primarily symptomatic and supportive, focusing on prevention of complications, rehabilitation, nutritional support, respiratory monitoring, and quality of life (15, 16).

This case highlights the importance of recognizing developmental regression as a major warning sign in pediatrics, even when initial metabolic testing and conventional neuroimaging are unrevealing. It also emphasizes the diagnostic value of genomic testing in ultra-rare neurodegenerative diseases and the role of reverse phenotyping in refining genotype–phenotype correlations. Overall, this report expands the clinical spectrum of PLA2G6-associated neurodegeneration by documenting a child with progressive regression and molecular support for INAD despite normal conventional brain MRI, reinforcing the need for integrated clinical, radiological, and molecular evaluation.

## 3 Conclusion

This case underscores the importance of considering PLA2G6-associated neurodegeneration in children with progressive neurodevelopmental regression, even when conventional brain MRI is normal. The combination of early motor and language regression, persistent hypotonia, bulbar dysfunction, and molecular identification of a homozygous PLA2G6 variant supports the diagnosis within the spectrum of infantile-onset PLA2G6-associated neurodegeneration. Importantly, this case illustrates the limitations of relying exclusively on neuroimaging findings and reinforces the role of early genomic testing in pediatric regression syndromes. Longitudinal neuroimaging, expert neuroradiological review, variant classification according to ACMG/AMP criteria, and parental segregation studies would further strengthen genotype–phenotype interpretation and guide genetic counseling.

## Data Availability

The original contributions presented in the study are included in the article/Supplementary Material, further inquiries can be directed to the corresponding author.
